# Comparison of five equations for estimating resting energy expenditure in Chinese young, normal weight healthy adults

**DOI:** 10.1186/2047-783X-17-26

**Published:** 2012-09-01

**Authors:** Zhi-yong Rao, Xiao-ting Wu, Bin-miao Liang, Mao-yun Wang, Wen Hu

**Affiliations:** 1 Department of Clinical Nutrition, West China Hospital of Sichuan University, Number 37 Guoxuexiang Road, Chengdu, 610041, China; 2 Center of Gastrointestinal Surgery, West China Hospital of Sichuan University, Number 37 Guoxuexiang Road, Chengdu, 610041, China; 3 Lung Function Laboratory of Department of Respiration, West China Hospital of Sichuan University, Number 37 Guoxuexiang Road, Chengdu, 610041, China

**Keywords:** Resting energy expenditure, Indirect calorimetry, Ideal body weight, Predictive equation

## Abstract

**Background:**

Most resting energy expenditure (REE) predictive equations for adults were derived from research conducted in western populations; whether they can also be used in Chinese young people is still unclear. Therefore, we conducted this study to determine the best REE predictive equation in Chinese normal weight young adults.

**Methods:**

Forty-three (21 male, 22 female) healthy college students between the age of 18 and 25 years were recruited. REE was measured by the indirect calorimetry (IC) method. Harris-Benedict, World Health Organization (WHO), Owen, Mifflin and Liu’s equations were used to predictREE (REEe). REEe that was within 10% of measured REE (REEm) was defined as accurate. Student’s *t* test, Wilcoxon Signed Ranks Test, McNemar Test and the Bland-Altman method were used for data analysis.

**Results:**

REEm was significantly lower (*P* < 0.05 or *P* < 0.01) than REEe from equations, except for Liu’s, Liu’s-s, Owen, Owen-s and Mifflin in men and Liu’s and Owen in women. REEe calculated by ideal body weight was significantly higher than REEe calculated by current body weight ( *P* < 0.01), the only exception being Harris-Benedict equation in men. Bland-Altman analysis showed that the Owen equation with current body weight generated the least bias. The biases of REEe from Owen with ideal body weight and Mifflin with both current and ideal weights were also lower.

**Conclusions:**

Liu’s, Owen, and Mifflin equations are appropriate for the prediction of REE in young Chinese adults. However, the use of ideal body weight did not increase the accuracy of REEe.

## Background

Resting energy expenditure (REE) is the largest component of total daily energy expenditure, accounting for 60% to 75% of total expenditure [1]. It represents the metabolic status of body cell mass in both the normal and pathological states. Measuring REE accurately is important for dietary therapy and nutrition support therapy. The metabolic cart is the standard procedure to measure REE. However, this procedure is time consuming, expensive, and usually unavailable because of the requirement for measuring respiratory exchange. More than 100 predictive equations have been developed [2-4] in order to circumvent this procedure and reduce the variability between measurements. These equations are based upon regressive analysis of body weight, height, sex, and age, or analysis of some independent variables, such as fat free mass, fat mass, body surface area, and total body potassium level [5]. However, these predictive equations are not always accurate in reflecting true REE, because they cannot completely reveal the relationship between the chosen variables and the actual resting energy expenditure in each individual [1,6]. Since most equations were developed from research in healthy subjects, it might not be appropriate to use them in patients. Recent studies in patients with different diseases demonstrated that predictive REE were about 10% higher than REE measured by indirect calorimetry (IC) [7,8]. In addition, it is not appropriate to apply predictive equations in all kinds of patients [9,10] because of increasing REE under pathological status [11,12]. It is also proposed that using ideal body weight instead of current body weight may increase the accuracy of the estimation in critically ill patients [13].

Most of the predictive equations were developed from studies in Caucasian people, and very few were from studies of Asians [14]. In order to determine the predictive equation most appropriate for the Chinese population, we carried out this study to compare REE calculated by five commonly used predictive equations with REE measured by IC.

For the majority of European public nutrition and clinical nutrition doctor or dietitian, to select one of the most appropriate, the minimum error prediction formula to predict an individual's energy requirements are the most important. In view of the present conclusions of the study on energy requirements is not unified, we hope that our study can provide a reference. [15,16].

## Methods

### Subjects

The subjects were undergraduates at Sichuan University, Chengdu, China. Inclusion criteria were: age between 18 and 25 years old, having healthy living habits (eating three punctual meals daily, sleeping and exercising regularly, no smoking, no drinking of alcohol). Exclusion criteria were: having diseases that affect the measurement of gas exchange and body metabolism, such as asthma, chronic obstructive pulmonary disease, pneumothorax, upper respiratory tract infection, fever, cancer, hyperthyroidism, diabetes mellitus, hypertension, kidney disease and so on. Forty-three subjects (age, 22.9 ± 2.0 years; 21 men (22.4 ± 1.0 years), and 22 women (23.4 ± 2.6 years)) were included in this study.

### Measurement of REE

REE was measured (REEm) in a thermo neutral environment by an open circuit indirect calorimetry (Ultima PFX system, SN: 218000305, Model: 790705–205, Medical Graphics Corp., St. Paul, Minnesota, USA) method with a mask belt, mouthpiece and nose clip. Subjects fasted overnight (12 hours) and rested for 30 minutes before the measurement. Measurements were taken between 8:00 am and 11:00 am. Gas analyzers were calibrated daily before measurement (reference gases (21% O_2_ and 79% N_2_), calibration gases (12% O_2_, 5% CO_2_ and 83% N_2_)). Pneumotach calibration was also performed daily by using a 3-liter calibration syringe. Measurements were carried out for at least 30 minutes. The steady state period was defined as a five minute period during which the change of average minute oxygen consumption (VO_2_) was less than 10% and the change of respiratory quotient (RQ) was less than 5% [17-20]. REEm was determined from VO_2_ and carbon dioxide production (VCO_2_) using the abbreviated Weir equation [21].

### Predicted REE

The following data were collected to estimate REE (REEe) from the Harris-Benedict (H-B) [2], World Health Organization (WHO) [22],[ Owen 23,^24^], [Mifflin 25] and [Liu’s 14] equations (Table 1): height, current body weight, age, and sex. Current body weight and height were measured by a Xiheng doctor’s type scale (Wuxi scale machine factory, Wuxi City, Jiangsu Province, China; model: RGZ-120). The ideal body weights of men and women were calculated using the Broca equation (ideal body weight (kg) = height(cm)-105) and the Broca reformative equation (ideal body weight (kg) = height(cm)-100) [26], respectively. For each predictive equation, two sets of REEe were generated from the two body weight types (ideal and current).

**Table 1:** Predictive equations to calculate REE

**Equations**	**age**	**REE (kcal/d)**	
		**Men**	**Women**
H-B	adult	66.47 + 13.75 × BW + 5.0 × Height-6.76 × Age	655.1 + 9.56 × BW + 1.85 × Height −4.68 × Age
WHO	18– 30y	15.057 × BW + 692.2	14.818 × BW + 486.6
Owen	adult	10.2 × BW + 875	7.18 × BW + 795
Mifflin	adult	10 × BW + 6.25 × Height-5 × Age + 5	10 × BW + 6.25 × Height-5 × Age-161
Liu’s	adult	13.88 × BW + 4.16 × Height-3.43× Age	13.88 × BW + 4.16 × Height-3.43 × Age-112.40

BW,body weight; WHO, World Health Organization; y, years.

### Ethical considerations and statistics

The study was approved by the Clinical Trial committee of our hospital and was performed in accordance with institutional guidelines and the Declaration of Helsinki. Statistical analysis was performed by the statistical package SPSS Version 13.0 (SPSS Inc.). REE values were expressed as mean ± SD. The degree of agreement between the predicted and measured REE was evaluated by Bland-Altman limits of agreement analysis [27,28]. The limits of agreement were defined as the mean difference ±1.96 standard deviations [29]. REEm were compared separately to two sets of REEe generated from ideal and current body weight using each equation. The estimated accuracy was defined as the percentage of the subjects whose REEe was within ±10% of REEm. Overestimation and underestimation were defined as > 10% and < 10% of REEm, respectively [1,30], and were reported as percentage of subjects. Differences between REEe and REEm were analyzed by Student’s *t* test for paired samples. The accuracy of the different predictive equations and of the two kinds of body weight were compared by using the Wilcoxon Signed Ranks Test and *χ*^2^ test (McNemar Test) for nonparametric samples. *P* < 0.05 was defined as statistically significant.

## Results

### Comparison between REEm and REEe and between REEe calculated by two sets of body weight

Forty-three subjects were recruited in this study. Physical characteristics of the subjects are presented in Table 2. The average body mass index (BMI) was 20.6 kg/m^2^. The means and standard deviations of REEm and REEe are presented in Table 3. The mean of REEm was 1,384.6 kcal/day in men and 1,094 kcal/day in women. There was no significant difference between REEm and REEe calculated by Liu’s-current body weight in male, or female, or total subjects, REEe by Liu’s-ideal body weight (Liu’s-s) in male, REEe by Owen-current body weight in male, or female, or total subjects, REEe by Owen-ideal body weight (Owen-s) in male or all subjects, and REEe by Mifflin-current body weight in men. REEm was significantly lower (*P* < 0.01) than REEe calculated by H-B-current body weight in male or female or total subjects, REEe by H-B-ideal body weight (H-B-s) in male, female or total, REEe by WHO-current body weight in male, female ( *P* = 0.019) or total, REEe by WHO-ideal body weight (WHO-s) in male, female, or total, REEe by Liu’s-s in female or total, REEe by Owen-s in female, REEe by Mifflin in female or total, and REEe by Mifflin-ideal body weight (Mifflin-s) in male ( *P* = 0.021) or female or total subjects.

**Table 2:** Physical characteristics of the subjects (x̄±s)

	**Men (number = 21)**	**Women (number = 22)**	**Total (number = 43)**
Age(years)	22.4 ± 1.0	23.4 ± 2.6	22.9 ± 2.0
Current body weight (kg)	64.2 ± 9.3^a^	49.0 ± 2.9^a^	56.5 ± 10.9
Ideal body weight (kg)	69.7 ± 8.7^a^	59.0 ± 5.8^a^	64.2 ± 9.1
Height(cm)	171.3 ± 7.9^a^	159.0 ± 5.8^a^	165.0 ± 9.2
BMI(kg/m^2^)	21.8 ± 2.2^a^	19.4 ± 1.7^a^	20.6 ± 2.3

^a^Significantly different between subjects of different sex (*P* < 0.01). BMI, body mass index.z.

**Table 3:** Comparison between measured REE (REEm) and predicted REE (REEe) (x̄±s)

**Methods**	**REE (kcal/d)**					
	**Men (number = 21)**	***P*value**^**a**^	**Women (number = 22)**	***P*value**^**a**^	**Total (number = 43)**	***P*value**^**a**^
IC	1384.6 ± 285.4	-	1094.6 ± 238.1	-	1236.3 ± 297.7	-
H-B	1657.1 ± 157.1	0.001	1309.0 ± 64.6	0.000	1479.0 ± 211.8	0.000
H-B-s	1589.1 ± 102.4	0.004	1404.2 ± 67.0	0.000	1494.5 ± 126.4	0.000
*P* value^b^	0.007	-	0.000	-	0.376	-
WHO	1659.4 ± 140.1	0.000	1213.3 ± 87.8	0.019	1431.2 ± 253.2	0.000
WHO-s	1741.2 ± 131.1	0.000	1360.8 ± 86.2	0.000	1546.6 ± 221.2	0.000
*P*value^b^	0.003	-	0.000	-	0.000	-
Liu’s	1452.7 ± 158.8	0.295	1149.5 ± 99.8	0.256	1297.6 ± 201.3	0.121
Liu’s-s	1527.9 ± 138.9	0.054	1287.6 ± 105.1	0.019	1405.0 ± 171.7	0.000
*P*value^b^	0.004	-	0.000	-	0.000	-
Owen	1347.2 ± 152.8	0.508	1147.1 ± 42.6	0.285	1244.9 ± 149.2	0.816
Owen-s	1386.3 ± 117.9	0.977	1218.6 ± 41.8	0.019	1300.5 ± 121.2	0.102
*P*value^b^	0.010	-	0.000	-	0.000	-
Mifflin	1495.8 ± 146.6	0.085	1206.2 ± 88.0	0.028	1347.6 ± 188.6	0.006
Mifflin-s	1550.0 ± 127.8	0.021	1305.7 ± 94.9	0.000	1425.0 ± 166.0	0.000
*P*value^b^	0.003	-	0.000	-	0.000	-

^a^*P* value of comparison between REEm and REEe; ^b^*P* value of comparison of REEe between ideal body weight and current body weight; end of the predictive equations’ name plus ‘-s’ means that REE was calculated by ideal body weight, otherwise by current body weight. H-B, Harris-Benedict; IC, indirect calorimetry; REE, resting energy expenditure; REEe, estimated REE; REEm, measured REE; WHO, World Health Organization.

REEe calculated by current and ideal body weight were also compared (Table 3). For each equation, the REEe calculated by ideal body weight were all significantly higher than those calculated by current body weight (*P* < 0.01) in both male and female groups. The only exception was the H-B equation in the male group. REEe calculated by ideal body weight was significantly lower than that calculated by current body weight ( *P* < 0.01) in this group.

### Accuracy of REEe

The estimation accuracy was defined as the percentage of the subjects whose REEe was within ±10% of REEm. Overestimation or underestimation was defined as >10% or <10% of REEm, respectively. Each REEe value was transformed into one of three categories, underestimated, accurate, and overestimated. The percentage of underestimated, accurate, and overestimated subjects for each equation is presented in Table 4. The Wilcoxon Signed Ranks Test was used to compare the accuracy rates between REEe-ideal body weight and REEe-current body weight. The results are presented in Table 5. In men, significantly different accuracy rates between two weight sets (*P* < 0.05) were found when Liu’s and Owen equations were used to calculate REEe. In women, all five equations had significantly different accuracy rates between the two weight sets ( *P* < 0.05 or *P* < 0.01).

**Table 4:** The accuracy rates of REEe calculated by current body weight and ideal body weight

**Methods**	**The state of accuracy**					
	**Underestimation**		**Accurateness**		**Overestimation**	
	**Men**	**Women**	**Men**	**Women**	**Men**	**Women**
H-B	4.76%	4.55%	28.57%	31.82%	66.67%	63.64%
H-B-s^a^	9.52%	0.00%	28.57%	22.73%	61.90%	77.27%
WHO	4.76%	9.09%	28.57%	50.00%	66.67%	40.91%
WHO-s	9.52%	4.55%	14.29%	27.27%	76.19%	68.18%
Liu’s	23.81%	22.73%	42.86%	45.45%	33.33%	31.82%
Liu’s-s	9.52%	4.55%	42.86%	27.27%	47.62%	68.18%
Owen	38.10%	22.73%	38.10%	45.45%	23.81%	31.82%
Owen-s	19.05%	9.09%	57.14%	40.91%	23.81%	50.00%
Mifflin	9.52%	18.18%	47.62%	36.36%	42.86%	45.45%
Mifflin-s	9.52%	4.55%	42.86%	27.27%	47.62%	68.18%

^a^End of the predictive equations’ name plus ‘-s’ means that REE was calculated by ideal body weight, otherwise by current body weight. H-B, Harris-Benedict; REEe, estimated resting energy expenditure; WHO, World Health Organization.

**Table 5:** Comparison of accuracy rates between REEe calculated by current body weight and REEe by ideal body weight

**Equations**	***P*value with Wilcoxon Signed Ranks Test**			***P*value with McNemar Test**		
	**Men**	**Women**	**Total**	**Men**	**Women**	**Total**
H-B	0.157	0.046	0.414	1.000	0.625	0.688
WHO	0.564	0.008	0.011	0.250	0.125	0.021
Liu’s	0.034	0.001	0.000	1.000	0.388	0.503
Owen	0.046	0.008	0.001	0.125	1.000	0.549
Mifflin	0.564	0.005	0.007	1.000	0.727	0.549

H-B, Harris-Benedict; REEe, estimated resting energy expenditure; WHO, World Health Organization.

The overestimated percentage amalgamated with underestimated percentage to form an inaccurate percentage, and was compared to the accurate percentage for each equation by the McNemar Test. In both sex groups, using ideal body weight did not decrease the inaccurate rates of REEe (*P* > 0.05). Among the five equations, the highest accuracy rate was produced by the Owen equation using ideal body weight in men (57.14%). Other relatively high accurate rates were from Liu’s and Mifflin equations using both ideal and current body weights in men, and from WHO, Liu’s, and Owen equations using current body weight in women(Table 4).

The overestimated rates of H-B and WHO using both ideal and current body weight were high, while this rate was low in Liu’s and Owen using current body weight (Table 4). The McNemar Test was used to compare the overestimated rates between two equations (Table 6). When using current body weight, the overestimated rates of H-B and WHO were both significantly higher than those of Liu’s and Owen (*P* < 0.01 or *P* < 0.05) in men, while in women, only the rate of the H-B equation was significantly higher than those of Liu’s and Owen equations ( *P* < 0.05). When using ideal body weight, the overestimated rates of the H-B and WHO equations were significantly higher than that of the Owen equation in men ( *P* < 0.01), and the rate of the WHO equation was significantly higher than that of Liu’s equation ( *P* < 0.05), while in women, only the rate of the H-B equation was significantly higher than that of the Owen equation ( *P* < 0.05).

**Table 6:** Comparison of overestimated rates between equations

**The pair of equations**	***P*value with McNemar Test**		
	**Men**	**Women**	**Total**
H-B & Liu’s	0.016	0.016	0.000
H-B & Owen	0.004	0.016	0.000
H-B-s & Liu’s-s^a^	0.375	0.500	0.125
H-B-s & Owen-s	0.008	0.031	0.000
WHO & Liu’s	0.016	0.500	0.004
WHO & Owen	0.004	0.500	0.001
WHO-s & Liu’s-s	0.031	1.000	0.031
WHO-s & Owen-s	0.001	0.125	0.000

^a^End of the predictive equations’ name plus ‘-s’ means that REE was calculated by ideal body weight, otherwise by current body weight. H-B, Harris-Benedict; WHO, World Health Organization.

### Bias and precision

**Figure 1:**
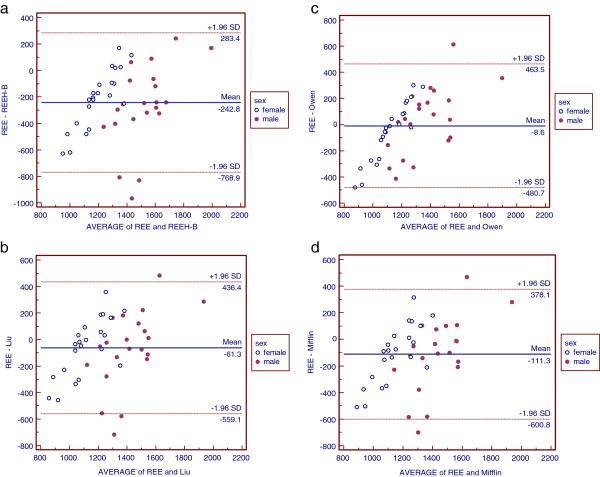
**Bland-Altman plots describing agreement between REEm and REEe from five equations and two body weight.** The differences of the REEm and REEe are plotted against their means. REEe, estimated resting energy expenditure; REEm, measured resting energy expenditure.

Bland-Altman limits of agreement analysis was used to determine the extent of error for each predictive equation by comparing it to REEm. For the entire study group, the lowest mean difference between REEm and REEe was found in the Owen prediction equation with current body weight (mean difference: -8.6 kcal/day); however, the extent of error was between −480.7 kcal and +463.5 kcal daily (See in Figure 1). This mean difference was also lower in Owen with ideal body weight and Liu’s with current body weight. The mean difference between REEe and REEm, limits of agreement, and standard error of the limits of agreement are presented in Table 7.

**Table 7:** Limits of agreement between REEm and REEe

**The equations for REEe**	**Mean difference REEe-REEm (kcal/day)**	**Limits of agreement (mean difference ±1.96SD) (kcal/day)**	**Standard error for the limits of agreement (95% confidence interval for the bias) (kcal/day)**	
			**Lower limits**	**Upper limits**
H-B	242.8	−283.4 to 768.9	160.2	325.4
H-B-s^a^	258.2	−252.3 to 768.8	178.1	338.4
WHO	194.9	−330.9 to 720.7	112.4	277.5
WHO-s	310.3	−248.6 to 869.3	222. 6	398.1
Liu’s	61.3	−436.4 to 559.1	−16.8	139.5
Liu’s-s	168.7	−373.1 to 710.5	83.6	253.8
Owen	8.6	−463.5 to 480.7	−65.5	82.7
Owen-s	64.2	−430.0 to 558.4	−13.4	141.8
Mifflin	111.3	−378.1 to 600.8	34.5	188.2
Mifflin-s	188.8	−333.0 to 710.6	106.9	270.7

^a^End of the predictive equations’ name plus ‘-s’ means that REE was calculated by ideal body weight, otherwise by current body weight. H-B, Harris-Benedict; REEe, estimated resting energy expenditure; REEm, measured resting energy expenditure; WHO, World Health Organization.

## Discussion

This study used an indirect calorimetry method to measure REE in 43 Chinese young healthy normal weight adults (age 22.9 ± 2.0 years) and compared the results with REEe generated from five predictive equations. The data suggest that most equations cannot accurately reflect REE in this study group. The source of the bias may be derived from equation limitations, the subjects’ characteristics, and measurement error. Several strategies were employed in this study to reduce the bias. The study group was representative of healthy Chinese undergraduates. REE, body weight, and height were all measured in well-controlled settings using current state of the art methodologies.

The Bland-Altman limits of agreement analysis showed that REEe generated from Owen and Mifflin equations agreed best with REEm (Figure 1). A recent study [31] also used the Bland and Altman method to analyze the agreement between REEe and REEm. It indicated that the Owen equation could be used to predict REE in young women with normal body weight. In our study, the average BMIs of men and women were 21.8 ± 2.2 kg/m^2^ and 19.4 ± 1.7 kg/m^2^, respectively, and the range was 18.5 kg/m^2^ to 24.9 kg/m^2^, except for one male subject who had a 27.1 kg/m^2^ BMI. In the original paper on the Mifflin equation, the authors selected 264 normal weight and 234 obese healthy subjects and found that body composition and body-weight distribution did not significantly affect REEe from various equations [25]. A recent study suggested that the Mifflin equation was valid in REE prediction in healthy Puerto Rican adults [32]. Another study in Belgian women showed that the Mifflin equation was a reliable tool to predict REE across a wide variety of body weight (BMI 18.5 to 50 kg/m^2^) [33]. In our study, we found that the Mifflin equation matched well with REEm. Also, we conclude that the Owen and Mifflin equations are more suitable to predict REE in this specific population.

Estimation accuracy was defined as the percentage of subjects whose REEe was within ±10% of REEm, considering it as a clinically relevant difference [4,30]. This error limit was accepted empirically because calorimetry measurement error is within ±5%. We also used ±10% of REEm to define accuracy; estimations above 10% of REEm were defined as overestimated, and those below 10% of REEm were defined as underestimated.

In our study, REEe were significantly higher than REEm. The exceptions were REEe from Liu’s, Liu’s-s, Owen, Owen-s, and Mifflin in men and Liu’s and Owen in women. This is not unexpected because both H-B and WHO equations were derived from research in subjects with a wide age range, but our study only included a limited number of young subjects. H-B was developed from research in healthy normal weight white men (n = 136, age 16 to 63 years) and women (n = 103, age 15 to 74 years), and had been adapted to be used in age groups from 21 to 70 years old [2]. The H-B equation is the most widely used equation in basal metabolic rate estimation. However, according to the original publication, the measurements were taken during the resting state, not under basal conditions [30]. In our study, we also measured REEm under resting conditions but REEe calculated by H-B equations using both current body weight and ideal body weight were still higher than REEm in both sexes. In a study about anorexia nervosa, the H-B equation overestimated REE; the reason might be that the patients’ BMIs were below normal [34]. Therefore, it can be accepted that the H-B equation overestimates REE in a young Chinese population.

The WHO equation was developed from research in young Europeans, most of whom were military and police recruits, with 45% of Italian descent [30]. In our study, the subjects were Chinese youth. This might explain the low accuracy rates of REE predicted by WHO in men (28.57%) in our study. However, the accuracy rate in women was 50.0%; maybe, the reason is that the subjects of this research comprised 2,279 men but only 247 women [30] and there was some bias in women *per se*. So the WHO equation is suitable for a European population, but not for Asians, especially, at least, not for Chinese men.

Liu’s equation was developed from research in a Chinese population. Previous studies have confirmed that Liu’s equation is the most appropriate one for predicting REE in healthy Chinese subjects [35]. In our study, the accuracy rates were 42.86% in men and 45.45% in women when using current body weight, and 42.86% in men when using ideal body weight. Compared with most other equations, these accuracy rates were higher. So, Liu’s equation is suitable for predicting REE in Chinese young subjects.

The Owen equation was derived from 44 healthy lean and obese women between 18 to 65 years old and 60 lean and obese men between 18 to 82 years old [23,24]. The Mifflin equation was derived from 498 healthy subjects (247 women, 251 men, 19 to 78 years old, among whom 264 were normal weight, and 234 obese) [25]. These two equations are commonly used in clinical practice. The accuracy rates of the Owen equation are 38.10% in men and 45.45% in women and the Mifflin equation had 47.62% and 36.36% accuracy rates in men and women, respectively (Table 4). The accuracy rates of these two equations are comparable to those of Liu’s. In addition, the Bland and Altman agreement analysis (Figure 1) indicated that these two equations had a lower extent of error in the study group. Moreover, in a study of Brazilian overweight and obese adults, the Mifflin equation had the best correlation with IC [36]. Thus, the Owen equation and the Mifflin equation could also be used to predict REE in Chinese young healthy normal weight adults.

Researchers normally use current body weight when applying the equations to predict REE, and they also define certain body types to meet the application range of the equations. However, in a real clinical setting, ideal body weight is often used in order to make subjects with abnormal body weight normal. Several studies showed that REE predicted by ideal body weight was more accurate than that predicted by current body weight [13,37,38]. In our study, by using Wilcoxon Signed Ranks Test, we found that current and ideal body weights generated obviously different accuracy rates of REEe. However, when we amalgamated the overestimated and underestimated subjects together as inaccurate, and analyzed the data, again using the McNemar Test, we found that there was no significant difference in the inaccuracy rates between two weight sets. In fact, REEe predicted by ideal body weight were higher than those predicted by current body weight. It seems that using ideal body weight did not increase the accuracy of REEe, except for the Owen equations in men. However, in a Brazilian study in patients with short bowel syndrome, researchers found that compared to current body weight, ideal body weight could increase the accuracy of REEe when using the H-B equation [39]. The difference between the two studies was probably due to the fact that patients with short bowel syndrome always had severe mixed-type malnutrition and low current body weight, so predicting REEe by using ideal body weight had considerable accuracy. However, in our study, ideal body weight was higher than current body weight in both sexes, while REEe from the current body weight was higher than REEm, so ideal body weight could not increase the accuracy of REEe. It may be that the normal average BMI of the subjects influenced the results. Some studies suggested using ideal body weight when predicting REE in obese subjects [40], while others preferredcurrent body weight in obese subjects [41]. Consequently, in our future study, we may need to classify the body type and research purpose in the prediction of REE by using ideal body weight.

We used the Bland and Altman method to assess the agreement between measured and predicted REE. Bland and Altman represented that, when comparing two clinical assessment methods, if neither of them can provide an unequivocally correct measurement, then the researchers should analyze the degree of their agreement instead of correlation or regression [42]. Still, many studies gave the correlation coefficient (*r*) between the results of two measurement methods as an indicator of agreement. Bland and Altman emphasized that *r* measures the strength of a relationship between two variables, not the agreement. In addition, a change in the scale of measurement does not affect the strength of correlation, but it affects the agreement [1]. For instance, if a REE predictive equation yields results exactly two times higher than REE obtained by IC, then correlation analysis would show one straight line with *r* =1, but the two measurements would not agree. Furthermore, the strength of the correlation between REEm and REEe increases when the ranges of REE are wider [43]. Regression analysis can be used to compare measurement methods because it attempts to predict the measured values (containing errors) from the observed values (considered without errors). For easier interpretation of the results, the same method should be used when assessing the agreement between two methods of clinical measurement [1].

## Conclusions

We found that H-B and WHO equations cannot be used to predict REE in Chinese young healthy normal weight adults. Liu’s, Owen, and Mifflin equations had higher accuracy rates in estimating REE. Bland and Altman analysis further suggested that Owen and Liu’s equations had a lower bias and agreed better with REEm. Taken together, we conclude that Liu’s, Owen and Mifflin equations can be used to predict REE in Chinese young healthy normal weight adults.

Ideal body weight is often used in the equations to predict REE in clinic settings and many studies proved that ideal body weight could increase the accuracy of predicted REE. However, in our study, ideal body weight did not increase the accuracy rates.

## Abbreviations

BMI, Body mass index; H-B equation, Harris-Benedict equation; IC, Indirect calorimetry; REE, Resting energy expenditure; REEe, Predicted resting energy expenditure; REEm, Measured resting energy expenditure; VO2, Oxygen consumption; VCO2, Carbon dioxide production; WHO, World Health Organization.

## Competing interests

The authors declare that they have no competing interests.

## Authors’ contributions

ZR was responsible for conceiving and designing the study, collecting and analyzing the data, and drafting the report. XW was responsible for revising the report. BL and MW were responsible for technical support during the measurement of REE. WH was responsible for interpreting the data and drafting the report. All authors read and approved the final manuscript.
